# Occurrence of plasmid mediated fluoroquinolone resistance genes amongst enteric bacteria isolated from human and animal sources in Delta State, Nigeria

**DOI:** 10.3934/microbiol.2021006

**Published:** 2021-02-03

**Authors:** EHWARIEME Daniel Ayobola, WHILIKI Onoriadjeren Oscar, EJUKONEMU Francis Ejovwokoghene

**Affiliations:** 1 Department of Microbiology, Faculty of Science, Delta State University, Abraka, Nigeria; 2 Department of Science Laboratory Technology, Delta State Polytechnic, Otefe-Oghara, Nigeria

**Keywords:** fluoroquinolones, enterobacteriaceae, animal husbandry

## Abstract

Plasmid mediated quinolone resistance (PMQR) is a public health challenge arising among other things, from indiscriminate use of the floroquinolones (FQr) prophylactically in animal husbandry. This study examines the occurrence of PMQR genes amongst enteric bacteria isolated from human and animal sources. A total of 720 (360 stool and 360 fish pond water/poultry litter) samples were examined for fluoroquinolone resistant (FQr) bacteria. Percentage FQr was generally higher among human isolates than isolates from animals. Proportion of PMQR amongst FQr isolates were (1.05 and 4.32) % for *E*. *coli* from human and animal sources. For *Salmonella* spp., *Shigella* spp., *Klebsiella* spp. and *Aeromonas* spp., percentages PMQR were 0.00 & 6.93, 0.00 & 6.38, 4.26 & 5.26 and 0.00 &3.03 for human and animal sources respectively, for the isolates. The PMQR genes: *qnr*A, *qnr* B, *qnr* S and *qep* A were 11, 15, 7 and 1 amongst a total of 1018 FQr and 29 PMQR isolates respectively. The aac (6′)–Ib-cr gene was not detected in this study. Approximate Plasmid bands of PCR amplicon for *qnr* A, *qnr* B, *qnr* S and *qep* A respectively were established. The proportion of PMQR genes especially among isolates from animal sources is of public health concern due to the higher possibility of a horizontal FQ resistance transfer to humans.

## 1. Introduction

Gastroenteritis, typhoid fever and a host of other gastro-intestinal tract (GIT) infections caused by bacteria of the Enterobacteriaceae are currently a significant cause of morbidity and mortality among children and adults in developing countries [1]. According to a 2004 World Health Organization (WHO) report, there is an annual incidence of 22 million cases of typhoid fever with over 216 thousand deaths per year [1]. Transmission of infection occurs mainly through consumption of contaminated food and water [2].

Enterobacteriaceae comprise a family of Gram-negative rod shaped bacteria. Some members exist as free-living organisms in the environment while some members are normal flora of the gastrointestinal tract of humans and animals [3]. Many are significant human and animal pathogens causing opportunistic infections following incorrect handling of food animals and fecal contamination of food and water [3],[4].

Antibiotics are natural, synthetic or semi-synthetic chemicals which in low concentration either inhibit the growth of or kill bacteria and are used to treat and prevent infections in humans and animals [5]. Quinolones are a class of synthetic, broad spectrum and bacteriocidal antibiotics that are active against both Gram-positive and Gram-negative bacteria including Enterobacteriacea and other intestinal pathogens and are used in human and veterinary medicine [6],[7]. The first quinolone in use was nalidixic acid. Fluoroquinolones (FQs) are quinolones with a fluorine atom at the C-6 position and include pefloxacin, ciprofloxacin, ofloxacin, norfloxacin, iomefloxacin, moxifloxacin and gemifloxacin. Fluoroquinolones are the drugs of choice for treatment of invasive gastrointestinal infections in adults worldwide. Quinolone exhibit their bacteriocidal activity by inhibiting DNA synthesis through inhibition of the enzyme topoisomerase IV and DNA gyrase and by causing breakage of bacterial chromosomes [7],[8].

Resistance to quinolones is being increasingly reported among humans and animals [9]. Antibiotic resistance occurs when bacteria are able to survive bacteriocidal or bacteriostatic effects of antibiotics it was once susceptible to [10]. The implication of quinolone resistance is that diseases caused by members of Enterobacteriacea become difficult to treat which in turn affects the economic and social life of those infected, resulting in high morbidity and mortality [11],[12].

Quinolones when used according to the manufacturer's directions should not result in resistance. However, factors contributing to quinolone resistance include: (i) It's misuse and overuse as a result of its effectiveness, less stringent regimen and inexpensiveness, over the counter availability and use of fake drugs [13] (ii) The use of quinolones at sub-therapeutic doses for purposes other than treatment of sick animals in livestock and aquaculture production [14] and (iii) Environmental pollution resulting from the discharge of untreated pond water containing antibiotic residue, antibiotic resistant bacteria and/or genes into the environment and the use of livestock manure for soil fertilization which may introduce antibiotic resistant bacteria into the environment from where resistance spreads to other bacteria [15].

Quinolone resistant bacteria may be transmitted to humans following direct exposure of humans to infected animals, their waste products and body fluids (such as blood, urines, feces, milk, saliva and semen) and occupational exposure of animal and food handlers. Antibiotic resistant bacteria and antibiotic resistant genes can also be transmitted to humans indirectly via contact with or consumption of contaminated food products (such as meat, eggs, milk and dairy products) containing antibiotic resistant bacteria or antibiotic resistance genes which has a far reaching effect than the direct transmission [16].

Bacteria acquire resistance to quinolones in two ways: chromosomal mutation in the genes *gyrA* and *parC* which encode the quinolone targets DNA gyrase and topoisomerase and acquisition of plasmid containing genes for quinolone resistance. Chromosomal mutation confers high–level of resistance to quinolones and it is transmitted vertically while plasmid mediated quinolone resistance (PMQR) confers low–level of resistance, it is transmitted horizontally among distantly related organisms making their spread much faster than that of chromosomal mutation [17].

Three major mechanisms are involved in PMQR: (a) limiting quinolone inhibition by qnr protein protection of drug targets. The following plasmid genes: *qnrA, qnrB, qnrC,qnrD, qnrS* and *qnrVC* code for proteins of the pentapeptide repeat family that protects DNA gyrase and topoisomerase IV from quinolone inhibition [18], (b) acetylation of the quinolone molecule by the variant aminoglucosideacetyltransferase *AAC(6′)–lb–cr* and (c) the quinolone specific efflux pump *QepA* [19].

Nigeria is not left out in the global, ravaging emergence of plasmid-mediated fluoroquinolone resistance (PMQR) that causes cases of treatment failure. Self-medication and abuse of antibiotics are common in Nigeria. Antibiotics are freely hawked in the open market while non-medically qualified persons make prescriptions. Quinolones also are used indiscriminately in livestock production and aquaculture. All these practices predispose quinolones to bacteria resistance. Therefore there is need for the knowledge of quinolone resistance in major towns in Delta State. This will assist the relevant agencies in formulating policies that will arrest the trend of antibiotic abuse and minimize the development of resistance.

The aim of this study was to survey for, and determine plasmid-mediated quinolone resistance determinants in enteric bacterial isolates of animal and human origin in Delta State, Nigeria.

## 2. Methods

### 2.1. Source and collection of samples

In this study, samples were obtained from two major sources namely, human (diarrhoeal stool of patients attending public hospitals, and those attending private hospital) and animal (poultry litter and fish pond water). A total of 720 samples were collected from three cities in Delta State. A total of 240 samples was collected from each city and comprise 60 stool samples from patients attending private hospitals, 60 stool samples from patients attending public hospitals, 60 fish pond water samples and 60 poultry litter samples. In each city, 20 mL of fish pond water was aseptically collected from 30 fish farms in sterile bijou bottles, dipped below the surface of the water, and transported to the laboratory in ice packs. 10 g each of poultry litter was aseptically collected in sterile bijou bottles containing peptone water from 30 poultry farms. 10g each of diarrhea stool samples were collected from 30 patients attending private and 30 from patients attending public hospitals and inoculated into sterile bijou bottle containing peptone water and selenite F broth. All samples were collected in duplicates and transported in ice packs to the Lahor research laboratory, Benin City, for analysis.

**Table 1.** Sample sources.

Location	Source of sample	Sample type	Number of samples [N = 720]
Human Samples			
Warri	Public Hospital (PBH)	Diarrhoeal stool	60
	Private Hospital (PRH)	Diarrhoeal stool	60
Sapele	Public Hospital (PBH)	Diarrhoeal stool	60
	Private Hospital (PRH)	Diarrhoeal stool	60
Ughelli	Public Hospital (PBH)	Diarrhoeal stool	60
	Private Hospital (PRH)	Diarrhoeal stool	60

Animal Samples			
Warri	Fish Farms	Fish Pond Water (FPW)	60
	Poultry Farms	Poultry Litter (PDR)	60
Sapele	Fish Farms	Fish Pond Water (FPW)	60
	Poultry Farms	Poultry Litter (PDR)	60
Ughelli	Fish Farms	Fish Pond Water (FPW)	60
	Poultry Farms	Poultry Litter (PDR)	60

### 2.2. Isolation, characterization and identification of enteric bacterial isolates

A Loop full from bijou bottles of all samples was streaked in triplicates onto prepared plates of Nutrient Agar (NA), MaCconkey Agar (MA) and Deoxycholate Citrate Agar (DCA) and incubated at 37 °C for 24 hours. Emerging colonies were subcultured for purification and subsequently identified based on morphology, gram reaction and biochemical characteristics, using the enterotube, according to guidelines of the Manual of Clinical Microbiology [20].

### 2.3. Standardization of bacteria isolates

Isolated colonies from pure culture plates were sub-cultured into peptone water, and incubated for 12 hours. Turbidity was then adjusted by dilution with sterile peptone water until visually comparable with a MacFarland's 0.5 standard. The MacFarland's 0.5 standard was prepared by adding Barium Chloride (BaCl) with Tetraoxosulphate VI acid (H_2_SO_4_). Standardized bacteria culture was referred to as bacteria stock solution, and was used for subsequent experiments.

### 2.4. Antimicrobial susceptibility test

All isolates were tested for susceptibility to the FQ antibiotics. This was carried out according to the standard disc diffusion technique as described by Clinical Laboratory Standard Institute (CLSI, 2014). Paper discs (AbtekBiologicals) containing the fluoroquinolones (FQs):Nalidixic acid NA-30 µg, Ciprofloxacin CPX-20 µg, Pefloxacin PEF-20 µg and Ofloxacin OFL-20 µg, were taken out of the refrigerator and allowed to equilibrate at room temperature. A sterile cotton swab was dipped into the respective bacterial stock solution, and excess fluid removed by pressing the swab against the wall of the tube. The entire surface of a Mueller-Hinton (MH) agar plate was then swabbed with the bacterial suspension, and allowed to dry for 15 min. Antibiotic discs were then layered aseptically, on the MH agar surface ensuring no air space forming between the disc and the plate.

Plates were then incubated at 37 °C overnight and the zones of inhibition (ZI) recorded after about 12 hours. Interpretation of results was based on guidelines of the Clinical and Laboratory Standards [21].

### 2.5. Plasmid curing experiment

The isolates showing resistance to FQ were subjected to plasmid curing experiments. Sodium dodecyl sulphate (SDS) was used as curing agent [22]. Graded concentrations (10 µg/mL to 1000 µg/mL) of curing agent was prepared, and loop full of standardized culture was seeded. Sub-lethal concentrations were tested for their ability to cure the bacteria of their resistance plasmids. Bacteria that lost their FQ resistance sequel to curing experiment were regarded as Plasmid-mediated FQ-resistant (PMQR) isolates. Isolates which on the other hand, retained their resistance were regarded as Chromosome-mediated FQ-resistant isolates. All PMQR isolates were subsequently subjected to plasmid extraction.

### 2.6. Plasmid DNA extraction experiment

This procedure was adopted from the Qiagen kit for rapid isolation of DNA. Ten colonies of pure culture of bacteria were emulsified in 1 mL Tris EDTA (TE) buffer in an Eppendrof tube. The tubes were spun at 5000 rpm for 5 minutes and the pellets were re-suspended in 100 µL TE-buffer. The tubes were boiled for 10 min at 100 °C. Then they were spun at 5000 rpm for 5 min (Eppendrof tubes were placed once). The supernatants were transferred to new clean Eppendrof tubes and the samples were stored at 2 °C until use.

### 2.7. Plasmid DNA characterization/gel electrophoresis

A 1.5% agarose (Bio-Rad, USA) in 0.75x Tris borate EDTA (TBE) buffer was prepared. (The agarose was dissolved by boiling the solution in microwave oven).A0.5 µg/mL Ethidium Bromide (EtBr) [Sigma-Aldrich LP, USA] was then added for staining the DNA molecules. The agarose-EtBr solution was poured into the gel tray of the electrophoresis apparatus containing the combs and allowed to set for 20 minutes. 5 µL of each DNA extract was loaded into the gel wells and 5 µL of 1 KB plus DNA molecular size marker (Invitrogen, USA) was loaded into one of the wells.

The electrophoresis was run at 120V for approximately 1 hour, 15 minutes and the gel was visualized on GelDoc system (BioRad, USA) and stored on disks as TIFF files.

### 2.8. PCR-based screening for PMQR genes

#### 2.8.1. *qnrA*, *qnrB*, and *qnrS*

All DNA extracts were subjected to a multiplex PCR for detection of the *qnr* genes. In multiplex PCR more than one target sequence are amplified by including more than one pair of primers in the reaction [23]. Multiplex PCR was used here due to the considerable savings of time and effort within the laboratory without compromising test utility.

Screening for the presence of *qnrA*, *qnrB*, and *qnrS* genes was carried out by modification of previously described PCR protocol [24]. The amplification was carried out using JumpStart™ REDTaq® ReadyMix™ PCR Reaction Mix and using specific primers for *qnrA*, to give a 516-bp product, for *qnrB*, to give a 469-bp product, and for *qnrS*, to give a 417-bp product [24]. The conditions were altered to 95 °C for 5 minutes then 30 cycles of 95 °C for 15 seconds, 55 °C for 15 seconds, and 72 °C for 40 seconds, then 72 °C for 4 minutes.

#### 2.8.2. PCR-Based Screening for *Aac(6′)-Ib-cr*

Screening for the presence of *aac(6′)-Ib* gene was carried out by modification of previously described PCR protocol [25]. The amplification was embarked upon using JumpStart™ REDTaq® ReadyMix™ PCR Reaction Mix and using specific primers for *aac(6′)-Ib*to give a 482-bp product [25]. The PCR conditions were altered to 95 °C for 5 minutes then 30 cycles of 95 °C for 15 seconds, 58 °C for 15seconds, and 72 °C for 40 seconds, then 72 °C for 4 minutes.

#### 2.8.3. PCR Based Detection of *Qep A*

Screening for the presence of the qep A gene was carried out as described by Yamane *et al*., 2008 with reagents as previously described, to give a 199 bp product [26] Base sequence of all primer sets used for this study is described below.

**Table 2.** Base sequence of all primer.

Primer Name	DNA Sequence (5′–3′)	Target site	Amplicon size (bp)
*aac(6′)-Ib-F*	ttgcgatgctctatgagtggcta	*aac(6′)-Ib*	482
*aac(6′)-Ib-R*	ctcgaatgcctggcgtgttt		
*qnrA-F* multiplex	atttctcacgccaggattg	*qnrA*	516
*qnrA-R* multiplex	gatcggcaaaggttaggtca		
*qnrB-F* multiplex	gatcgtgaaagccagaaagg	*qnrB*	469
*qnrB-R* multiplex	acgatgcctggtagttgtcc		
*qnrS-F* multiplex	acgacattcgtcaactgcaa	*qnrS*	417
*qnrS-R* multiplex	taaattggcaccctgtaggc		
*qepA-F*	gcaggtccagcagcgggtag	*qepA*	199
*qepA-R*	cttcctgcccgagtatcgtg		

### 2.9. Statistical analysis

Comparison of zones of inhibition of the various fluoroquinolones was determined using the one-way Analysis of Variance, while comparison between zones of inhibition of FQs on isolates from human and animal sources was done using the student's t-test. Proportions of occurrences and charts were done by descriptive statistics, all using the spss 16.0 package.

## 3. Results

Five different enteric bacterial species, *Escherichia coli, Salmonella spp., Shigella spp., Klebsiella spp*. and *Aeromonas spp*. were isolated in varying proportions. The overall population of the isolates stood at 1,964 with the prevalence rate shown in Table 3. *E.coli* alone, accounted for about one-third of the entire isolates from each of the sources with a range of 28.2–44.7%. *Klebsiella spp*. was the least encountered among all isolates, ranging from 6.8% to 12.3%. Generally, the population of isolates varied from one source to another, with stool from patients attending private hospitals having the least (396), and fish pond water having the highest number of isolates (563). The isolates were recovered more from the animal sources than the human sources.

**Table 3.** The number of enteric bacterial isolates from sampling sites.

Species	Number of isolates from:				All samples
	Public Hospital (PBH)	Private Hospital (PRH)	Poultry Droppings (PDR)	Fish-pond Water (FPW)	
*E.coli*	168 (33.6)	177 (44.7)	165 (32.7)	159 (28.2)	669 (34.1)
*Salmonella spp*.	126 (25.2)	87 (22.0)	177 (35.0)	98 (17.4)	488 (24.8)
*Shigella spp*.	131 (26.2)	76 (19.2)	46 (9.1)	68 (12.1)	321 (16.3)
*Klebsiella spp*.	44 (8.8)	27 (6.8)	61 (12.1)	69 (12.3)	201 (10.2)
*Aeromonas spp*.	31 (6.2)	29 (7.3)	56 (11.1)	169 (30.0)	285 (14.5)
Total isolates	500	396	505	563	1964

**Figure 1.**
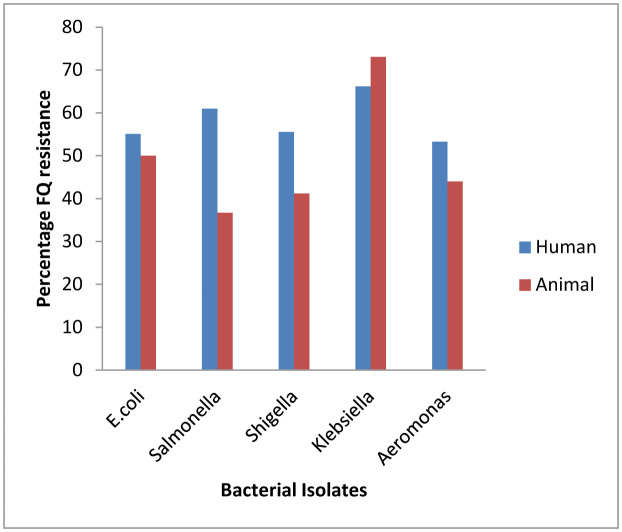
Relative proportion of fluoroquinolone resistance among isolates from human and animal sources.

For *Escherichia coli*, the percentage of FQ resistance was higher with isolates from patients attending public hospital (73.2), than from private hospitals (37.9). Also, the percentage FQ resistance was higher with isolates from poultry litter (61.2) than for isolates from fish pond water (38.4). This trend was equally observed with all isolates except *Klebsiella spp*. with higher FQ resistance observed for isolates from patients attending private hospital than those from public hospitals (Figure 1). Generally however, percentage FQ resistance tended to be higher with isolates from human sources, than animal sources.

Statistical analysis by ANOVA shows that there was significant differences (F: 170.944–613.302; P: 0.000) in the zones of inhibition (Table 4) however, Post-hoc test reveals that the differences exist in the resistance pattern between Nalidixic acid and Ofloxacin for isolates obtained from Public hospitals, Private hospital and Fish pond water. Only for *E.coli* isolates from poultry litter, did resistance pattern show significant difference for every single FQ tested

A similar observation was recorded for *Salmonella spp*. There was a significant difference in the resistance pattern of isolates from all sources to the FQs. However, for isolates from poultry litter, no significant difference existed in the resistance pattern to Ciprofloxacin and Pefloxacin (Table 4). Isolates of *Shigella spp*. from Public and Private Hospitals, as well as those from Poultry droppings and Fish pond water were significantly different in their zones of inhibition to all FQs tested (Table 4). Furthermore, *Klebsiella spp*., from human sources was significantly different to all tested FQs but isolates from animal sources displayed similar resistance pattern to Ciprofloxacin and Pefloxacin (Table 4). However, there was generally a significant difference in zones of inhibition of the tested FQs.

For isolates of *Aeromonas spp*. from Public hospital, they were significantly different (P < 0.05) in their resistance to the tested FQs. Similar observation was recorded for isolates from Fish pond water and Poultry droppings. However, resistance to Ciprofloxacin and Pefloxacin were not significantly different among isolates obtained from patients attending private hospital (Table 4).

Generally, the observation was that resistance to the various FQs by all bacterial isolates were significantly different irrespective of their sources, but there tended to be a similarity in the resistance pattern to Ciprofloxacin and Pefloxacin. While resistance to Nalidixic acid and Ofloxacin was always significantly different, resistance pattern to Ciprofloxacin and Pefloxacin were often similar.

One major aim of this study was to compare resistance of isolates from animals, with those from humans. All comparisons for susceptibility testing were performed using the t test with the SPSS version 16.0 at a 5% probability level (Table 5).

**Table 4.** Susceptibility of isolates to the Fluoroquinolones.

Sources	Zones of inhibition (mean ± SD) mm				F value	Sig.
	Nalidixic acid	Ciprofloxacin	Pefloxacin	Ofloxacin		
*Escherichia coli*
(PBH)	4.2 ± 0.59^a^	8.4 ± 0.94^b^	8.9 ± 1.40^b^	13.1 ± 2.70^c^	613.302	0.000
(PRH)	4.1 ± 0.64^a^	8.8 ± 1.39^b^	9.0 ± 1.51^b^	11.5 ± 2.50^c^	231.306	0.000
(PDR)	4.4 ± 0.70^a^	8.0 ± 1.14^b^	8.8 ± 1.42^c^	12.1 ± 2.81^d^	341.371	0.000
(FPW)	4.3 ± 0.73^a^	8.2 ± 1.68^b^	8.6 ± 2.26^b^	12.7 ± 2.87^c^	170.944	0.000
*Salmonella* sp.
(PBH)	4.2 ± 0.77^a^	8.2 ± 1.69^b^	8.8 ± 1.96^b^	11.8 ± 2.36^c^	262.940	0.000
(PRH)	4.2 ± 0.51^a^	8.3 ± 1.56^b^	9.0 ± 1.90^b^	11.8 ± 3.42^c^	94.018	0.000
(PDR)	3.9 ± 0.76^a^	8.2 ± 1.42^b^	8.9 ± 1.84^c^	13.4 ± 2.92^d^	271.532	0.000
(FPW)	3.5 ± 0.88^a^	8.6 ± 1.90^b^	8.7 ± 1.61^b^	12.7 ± 2.65^c^	144.776	0.000
*Shigella* sp.
(PBH)	3.9 ± 0.84^a^	8.0 ± 1.22^b^	9.4 ± 2.12^c^	12.2 ± 2.32^d^	300.895	0.000
(PRH)	4.5 ± 0.62^a^	7.5 ± 0.79^b^	9.3 ± 2.18^c^	12.2 ± 2.38^d^	137.107	0.000
(PDR)	2.5 ± 0.43^a^	6.3 ± 1.73^b^	8.4 ± 1.54^c^	11.7 ± 3.90^d^	82.472	0.000
(FPW)	2.7 ± 0.45^a^	5.9 ± 0.37^b^	9.2 ± 2.17^c^	13.6 ± 2.51^d^	136.899	0.000
*Klebsiella* sp.
(PBH)	3.2 ± 0.79^a^	5.2 ± 0.96^b^	9.5 ± 1.92^c^	13.0 ± 2.84^d^	163.729	0.000
(PRH)	3.5 ± 0.61^a^	5.4 ± 0.79^b^	9.6 ± 2.21^c^	11.2 ± 3.71^d^	49.489	0.000
(PDR)	3.5 ± 0.70^a^	8.2 ± 1.41^b^	8.5 ± 1.55^b^	12.5 ± 3.42^c^	158.643	0.000
(FPW)	3.4 ± 0.66^a^	7.6 ± 1.33^b^	8.4 ± 1.75^b^	13.3 ± 2.99^c^	220.856	0.000
*Aeromonas* sp.
(PBH)	3.0 ± 0.69^a^	7.8 ± 1.25^b^	10.2 ±2.12^c^	13.5 ± 3.00^d^	105.466	0.000
(PRH)	3.5 ± 0.52^a^	9.4 ± 1.64^b^	9.7 ± 2.48^b^	13.0 ± 2.37^c^	46.767	0.000
(PDR)	3.3 ± 0.61^a^	5.2 ± 0.93^b^	8.8 ± 1.72^c^	13.5 ± 2.82^d^	205.346	0.000
(FPW)	3.3 ± 0.65^a^	5.3 ± 0.95^b^	7.8 ± 1.48^c^	13.2 ± 3.21^d^	362.324	0.000

(Means with similar alphabets on same row are not significantly different)

The zones of inhibition of the FQs were compared for *E.coli* obtained from human and animal sources. It was observed that there was no significant difference in the zones of inhibition exerted by OFL and PEF but the mean zones of inhibition of NA and CPX on *E.coli* isolates were significantly different for isolates from human and animal sources (Table 5).

For *Salmonella* isolates, there was a significant difference in the zones of inhibition by NA and OFL in respect of isolates from human and animal sources (Table 5). However, no significant difference was observed in the mean zones of inhibition by CPX and PEF on isolates from human and animal sources. Furthermore, the mean zones of inhibition by NA, PEF and CPX on *Shigella sp*. isolates from human and animal sources, were significantly different, but no significant difference existed in the resistance to OFL by *Shigella* isolates from human and animal sources (Table 5).

**Table 5.** Comparison of FQ resistance of isolates from human and animal sources.

Fluoroquinolone	Zones of inhibition (mean±SD) mm		P value
	Human	Animal	
*E.coli*
Nalidixic Acid	4.20 ± 0.61	4.39 ± 0.71	0.007
Ciprofloxacin	8.56 ± 1.13	8.05 ± 1.37	0.000
Pefloxacin	8.90 ± 1.45	8.70 ± 1.78	0.250
Ofloxacin	12.50 ± 2.72	12.32 ± 2.84	0.707
*Salmonella* sp
Nalidixic Acid	4.21 ± 0.69	3.76 ± 0.82	0.000
Ciprofloxacin	8.25 ± 1.65	8.36 ± 1.61	0.502
Pefloxacin	8.86 ± 1.94	8.84 ± 1.76	0.975
Ofloxacin	11.80 ± 2.74	13.16 ± 2.84	0.000
*Shigella* sp.
Nalidixic Acid	4.13 ± 0.82	2.59 ± 0.44	0.000
Ciprofloxacin	7.87 ± 1.12	6.14 ± 1.39	0.000
Pefloxacin	9.38 ± 2.13	8.70 ± 1.82	0.041
Ofloxacin	12.17 ± 2.33	12.40 ± 3.53	0.682
*Klebsiella* sp.
Nalidixic Acid	3.33 ± 0.74	3.41 ± 0.68	0.504
Ciprofloxacin	5.29 ± 0.89	7.91 ± 1.39	0.000
Pefloxacin	9.52 ± 2.02	8.43 ± 1.64	0.002
Ofloxacin	12.31 ± 3.31	12.91 ± 3.22	0.309
*Aeromonas* sp.
Nalidixic Acid	3.20 ± 0.66	3.31 ± 0.63	0.436
Ciprofloxacin	8.31 ± 1.58	5.27 ± 0.94	0.000
Pefloxacin	10.03 ± 2.22	8.11 ± 1.61	0.000
Ofloxacin	13.36 ± 2.77	13.31 ± 3.08	0.928

Resistance to CPX and PEF were significantly different for human and animal isolates while resistance to NA and OFL were not, for *Klebsiella* isolated from human and animal sources (Table 5). Resistance by *Aeromonas* from human and animal sources to CPX and PEF, were significantly different but resistance to NA and OFL were not, for isolates from human and animal sources (Table 5).

Generally, the CPX and PEF resistance pattern exhibited by isolates from human and animal sources tended to be always similar except for *E.coli*. Furthermore, resistance of human and animal isolates to OFL were always not significantly different except for *Salmonella* isolates from human and animal sources that were significantly different with human isolates exhibiting significantly higher resistance.

The proportion of plasmid mediated quinolone resistance among all isolates of *E.coli* is presented in Table 6. Isolates from animal sources had a higher occurrence of plasmid mediated resistance than isolates from human sources. Furthermore, the proportion of PMQR *E.coli* among FQ resistant *E.coli* equally showed animal isolates having a higher proportion in comparison with isolates from human sources (Table 6). This observation was generally similar among all enteric bacteria isolates of *Salmonella spp*. (Table 7), *Shigella* spp. (Table 8), *Klebsiella* spp. (Table 9) and *Aeromonas* spp. (Table 10). Generally, all bacterial isolates from animal sources showed higher proportion of plasmid-mediated quinolone resistance than isolates from human sources.

**Table 6.** Proportion of Plasmid Mediated Quinolone Resistance in all *E.coli* isolates from human and animal sources and Proportion of Plasmid Mediated Quinolone Resistance in Quinolone resistant *E.coli* isolates from human and animal sources.

Sources		Total number of isolates/FQr	Number of PMQR isolates	PMQR Proportion (%) Per total isolates/Per FQr	
Animal	Poultry Droppings	165/101	5	3.03	4.95
	Fish Pond Water	159/61	2	1.26	3.28
	TOTAL	324/162	7	2.16	4.32
Human	Public Hospital	168/123	1	0.60	0.81
	Private Hospital	177/67	1	0.56	1.49
	TOTAL	345/190	2	0.58	1.05

**Table 7.** Proportion of Plasmid Mediated Quinolone Resistance in all *Salmonella* isolates from human and animal sources and Proportion of Plasmid Mediated Quinolone Resistance in Quinolone resistant *Salmonella* isolates from human and animal sources.

Sources		Total number of isolates/FQr	Number of PMQR isolates	PMQR Proportion (%) Per total isolates/Per FQr	
Animal	Poultry Droppings	177/65	4	2.26	6.15
	Fish Pond Water	98/36	3	3.06	8.33
	TOTAL	275/101	7	2.55	6.93
Human	Public Hospital	126/87	0	0.00	0.00
	Private Hospital	87/43	0	0.00	0.00
	TOTAL	213/130	0	0.00	0.00

**Table 8.** Proportion of Plasmid Mediated Quinolone Resistance in all *Shigella* isolates from human and animal sources and Proportion of Plasmid Mediated Quinolone Resistance in Quinolone resistant *Shigella* isolates from human and animal sources.

Sources		Total number of isolates/FQr	Number of PMQR isolates	PMQR Proportion (%) Per total isolates / Per FQr	
Animal	Poultry Droppings	46/29	1	2.17	3.45
	Fish Pond Water	68/18	2	2.94	11.1
	TOTAL	114/47	3	2.63	6.38
Human	Public Hospital	131/77	0	0.00	0.00
	Private Hospital	76/38	0	0.00	0.00
	TOTAL	207/115	0	0.00	0.00

**Table 9.** Proportion of Plasmid Mediated Quinolone Resistance in all *Klebsiella* isolates from human and animal sources. and Proportion of Plasmid Mediated Quinolone Resistance in Quinolone resistant *Klebsiella* isolates from human and animal sources.

Sources		Total number of isolates/FQr	Number of PMQR isolates	PMQR Proportion (%) Per total isolates/Per FQr	
Animal	Poultry Droppings	61/48	4	6.56	8.33
	Fish Pond Water	69/47	1	1.45	2.13
	TOTAL	130/95	5	3.85	5.26
Human	Public Hospital	44/28	1	2.27	3.57
	Private Hospital	27/19	1	3.70	5.26
	TOTAL	71/47	2	2.82	4.26

**Table 10.** Proportion of Plasmid Mediated Quinolone Resistance in all *Aeromonas sp*. isolates from human and animal sources. and Proportion of Plasmid Mediated Quinolone Resistance in Quinolone resistant *Aeromonas* isolates from human and animal sources.

Sources		Total number of isolates/FQr	Number of PMQR isolates	PMQR Proportion (%) Per total isolates/Per FQr	
Animal	Poultry Droppings	56/31	2	3.57	6.45
	Fish Pond Water	169/68	1	0.59	1.47
	TOTAL	225/99	3	1.33	3.03
Human	Public Hospital	31/21	0	0.00	0.00
	Private Hospital	29/11	0	0.00	0.00
	TOTAL	60/32	0	0.00	0.00

With the exception of *Salmonella* isolates from animal sources, which had the highest proportion of plasmid mediated quinolone resistance among FQ resistant isolates (Table 7), *Klebsiella* spp. generally had the highest occurrences of plasmid mediated quinolone resistance from human sources (Table 9). Generally, there were no plasmid resistant isolates of *Salmonella* (Table 5), *Shigella* (Table 8) and *Aeromonas* spp (Tables 10) from human sources.

**Figure 2.**
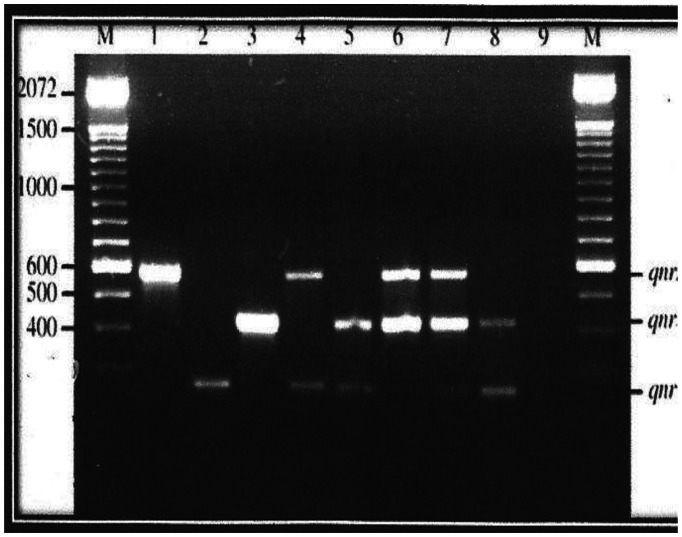
Plasmid bands and their approximate molecular weights of Plasmid-Mediated Resistant Isolates.

**Figure 3.**
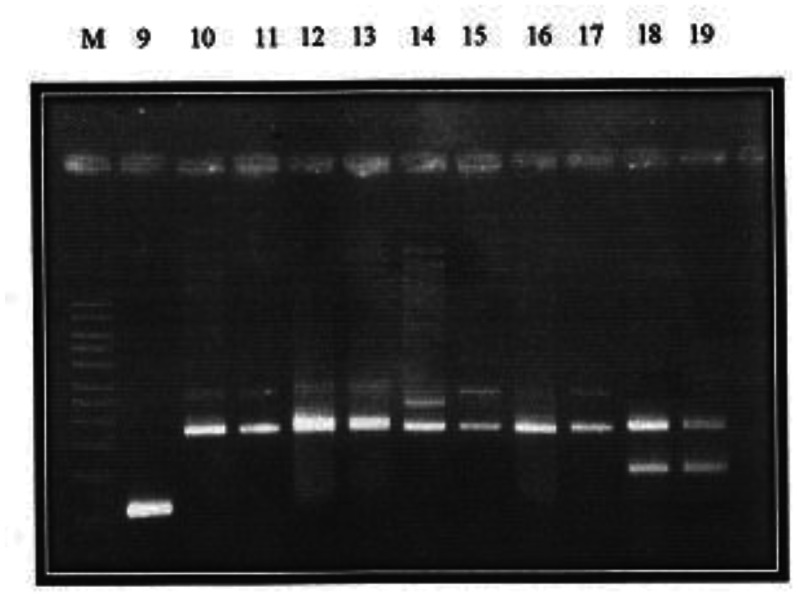
Plasmid bands and their approximate molecular weights of Plasmid-Mediated Resistant Isolates. *Qnr A*: Lanes 1, 4, 6, 7, 10, 11, 12, 13, 14, 15, 17; *Qnr B*: Lanes 3, 5, 6, 7, 8, 10, 11, 12, 13, 14, 15, 16, 17, 18, 19; *Qnr S*: Lanes 2, 4, 5, 7, 8, 18, 19; *Qep A*: Lane 9.

While the *aac*(6′)-Ib-cr gene was not detected in the present study, the *qnr*S gene had the highest occurrence, both among the FQ-resistant and PMQR (Table 11, Figures 2 and 3) isolates. The only qepA gene detected was among isolates of *Salmonella spp.*, and it was obtained from poultry sample.

**Table 11.** PCR detection and proportion of the plasmid-mediated quinolone resistance genes in fluoroquinolone resistant isolates and PCR detection and proportion of the plasmid-mediated quinolone resistance genes in plasmid-mediated fluoroquinolone resistant isolates.

Quinolone resistant/PMQR isolates	Number of isolates with:				
	qnrA	QnrB	qnrS	qepA	aac(6′)-Ib-cr
*Aeromonas* sp. (n = 131/3)	2	2	0	0	0
*E.coli* (n = 352/9)	4	3	3	0	0
*Klebsiella* sp. (n = 142/7)	2	3	0	0	0
*Salmonella* sp. (n = 231/7)	2	5	3	1	0
*Shigella* sp. (n = 162/3)	1	2	1	0	0
Total (N = 1018/29)	11	15	7	1	0

## 4. Discussions

The results of the study revealed that *E*. *coli* was the dominant species among the enteric organisms isolated from both animal and human sources. This observation agrees with previous studies and is quite significant especially for fish pond water samples. The presence of *E.coli* is indicative of fecal pollution [27], due to the fact that the gastro-intestinal tract is the natural habitat of the organism. Hence, this suggests a possible contamination of the fish pond with human or animal excreta. This was the case with a good number of these fish ponds surveyed, that were integrated with poultry. It is of public health concern because, if products of aquaculture from such ponds are not properly boiled before consumption, could pose health risk to the consumer [28].

In poultry litter, the greatest prevalence was however *Salmonella* sp. This again, could cross-contaminate fish pond water when used in pond fertilization and even egg shells, resulting in infection when proper sanitary conditions are not observed. From previous researches, *Salmonella* is more frequently isolated from chicken litter or fecal samples as compared to other pathogens investigated [29],[30].

The observation in this study was that for all isolates, the percentage resistance was higher among isolates from public hospital than private hospitals. Individuals visiting public hospitals tend to be those of the low income category, who equally engage in self-medication more than the higher income category. Self-medication is one major form of antibiotic misuse which is a cause of resistance emergence. Also, the percentage FQ resistance was higher among isolates from poultry litter, than isolates from fish pond water. From market surveys conducted during this study, antibiotics sold and used in aquaculture, is largely tetracycline but those sold and used in poultry, were quite varied and more numerous. The FQs are well represented in almost every additive administered to poultry. This indiscriminate prophylactic use of the FQs in poultry may be responsible for the high resistance among isolates from poultry. This observation is an emerging concern [27].

A comparison of resistance trend among isolates from human and from animals, were also conducted. Percentage FQ resistance tends to be higher among human isolates than animal isolates. This came as a surprise, because of the initial observation that misuse and abuse is higher in animal husbandry. This research has however proved otherwise. Individuals still indulge in antibiotic misuse and abuse, more than the prophylactic use of antibiotics in animal farming. There is paucity of information on such comparative study. With increasing antibiotic abuse, and non-adherence to prescription regimen, we tend to perpetually place the bacteria steps ahead of man in the resistance development tussle.

A further comparison of the resistance patterns to the various test FQs was undertaken. Resistance to all FQs was significantly different. A similar resistance pattern was however recorded for Ciprofloxacin and Pefloxacin. These drugs are commonly marketed with the trade names Ciprotab/ Ciprocin and Peflotab respectively. They are quite affordable and accessible and as such highly prone to abuse. On the other hand, Ofloxacin, with trade name Tarivid, is costlier less affordable and accessible hence its limited abuse. This may explain the greater susceptibility of the organisms to Ofloxacin when compared to other FQs. Previous studies has focused more on Ciprofloxacin [31],[32],[33], as this seems to be the most common of all FQs.

The involvement of plasmids has further compounded the widespread occurrence of drug resistance [34]. The FQs have suffered same fate, as reports from other nations confirm emergence of Plasmid Mediated Quinolone Resistance (PMQR) among bacteria [24],[34]. In the present study, PMQR was equally recorded among isolates from the Nigerian Niger-delta. Among the FQs studied, resistance to Ofloxacin by isolates was largely chromosome mediated. The PMQR phenomenon was more observed with Ciprofloxacin and Pefloxacin, with resistance being lost sequel to plasmid curing in line with previous studies [35],[36].

In line with the objective of this study, comparison of PMQR among isolates from animal and human sources was determined. As observed earlier, FQ resistance was more among human isolates than animal isolates. In the case of plasmid involvement with FQ resistance however, there was a reversal in the trend as PMQR was higher among animal isolates than human isolates as observed in a previous study [37]. The possible explanation for this observation lies in the mode of spread and dissemination of resistance genes. While chromosomally mediated resistance is disseminated basically vertically from parents to offspring, plasmid-mediated resistance is disseminated both vertically and horizontally from one bacterium to another, by conjugation, transformation and transformation. These mechanisms tend to be enhanced by the microbial pool prevalent in animal settings.

Implication of this observation is that, FQ resistance will spread faster among humans if organisms harboring FQ resistance plasmids are transferred by zoonotic cross-contamination, to man. The relative ease with which antibiotic resistance borne on plasmids are spread, [24],[34] indicates that FQ resistance will spread rapidly within the animal pool and eventually cross migrate to man. Humans are hence at a risk of acquiring FQ resistance either directly, or indirectly even in the absence of antibiotic abuse.

In this study, the *aac(6′)-Ib-cr* determinant was not detected at all. This is in contrast with what has been observed in other studies; the prevalence of *aac(6`)-Ib-cr* was 11.3% (62/549) among ciprofloxacin-and/or tobramycin-resistant *E. coli* and *Klebsiella spp*. clinical isolates from Canada [38] and 9.9% (36/365) among Extended Spectrum Beta-lactamase (ESBL)-producing *E. coli* and *K. pnumoniae* isolates from six provinces in China [39] while it was 51% among ciprofloxacin-resistant clinical isolates of *E. coli* isolated from Shanghai, China [34]. This gene, a variant of the aminoglycoside acetyltransferase, is responsible for modification of the FQ and its subsequent inactivation [33],[40]. Usually, it confers resistance on Ciprofloxacin only, since only Ciprofloxacin among the other FQs are the only compounds with an un-substituted piperazinyl group [40]. This may account for the observation in this study that resistance to Ciprofloxacin alone did not occur. All FQ resistance was jointly associated with at-least two of the quinolones under study.

On the other hand, the occurrence of the quinolone efflux pump protein *qep*A was very low in this study. Experimentally, the *qep*A protein does not alter the MICs of ampicillin, erythromycin, kanamycin or tetracycline, but it does decrease susceptibility to norfloxacin, enrofloxacin and ciprofloxacin by up to 64-fold [41]. This may be attributed to the fact that the *qep* determinant is only emerging when compared to other determinants. Indeed previous studies also showed a low prevalence of this gene where it occurs [26],[42],[43]. There is mounting information about the epidemiology of the newly discovered PMQR pump *qep*A. In the present study, only one (0.10%) *qep*A was identified among the FQ-resistant population and, that was amongst *Salmonella sp*. A survey conducted in Japan found *qepA* in 2 (0.3%) of 751 *E. coli* isolates collected from 140 hospitals between 2002 and 2006 [26]. A second large survey was done by PCR in France. A single *E. coli* isolate among 121 (0.8%) ESBL-positive *Enterobacteriaceae* strains isolated in 2007 was positive for a variant named *qepA2* [44]. In a study of pig farms in China, *qepA* was found in 28 of 48 (58.3%) aminoglycoside resistant methyl transferase, *rmtB*-positive *E.coli* isolates [45]. A follow-up study from the same region in China tested for *qepA* among ceftaxidime-resistant isolates of *Enterobacteriaceae. qepA* was found in 16 of 101 (15.8%) isolates, including, for the first time, *K. pneumoniae* and *E. cloacae* [46]. Few recently published studies indicated a broad distribution of the gene. A survey of 461 isolates of *Enterobacteriacea* in South Korea found *qepA* in one isolate from 2005 [47]. *qepA* has also been found in the United Kingdom. Two additional studies screened isolates from Seoul, South Korea, for *qepA*. Four clonally unrelated strains of 621 (0.6%) *E. coli* bloodstream isolates were found to be positive in one study [48], and two *E. aerogenes* isolates of 223 (0.9%) *E. cloacae*, *E. aerogenes*, *C. freundii*, and *Serratia marcescens* isolates with reduced susceptibility to quinolones were *qepA* positive in the second survey [49]. *qepA* was not found in a large survey of non-Typhi *Salmonella enterica* isolates collected in the United States from 1996 to 2006 [50].

Although the findings indicated a low prevalence, the previous studies cited showed that this determinant was found transferable in most cases. This suggests that its presence in the Niger-delta could quickly be amplified due to vertical and horizontal dissemination.

This study also revealed the low occurrence of the *qnr* determinants. A similar low occurrence of *qnr* was also reported in Denmark where only 1.63% (2/122) of nalidixic acid-resistant *E. coli* isolates was *qnr*-positive [51]. Low prevalence of *qnr* genes has also been reported from France and Canada. In France, the prevalence of *qnr* genes was 1.6% (2/125) among ESBL producing *E. coli* and *Klebsiella* spp. isolates [44],[52] while in Canada only about 1% (5/550) of ciprofloxacin and/or tobramycin resistant *E. coli* and *Klebsiella* spp. isolates were *qnr*-positive [38]. Nevertheless, higher prevalence has also been detected in other parts of the world such as Spain (5%) [53], China (8%) [39] and the United States (15%) [24]. Furthermore, investigations in China showed that *qnr, aac(6′)-Ib-cr, qepA*, and *oqxAB* genes were detected in 5.7%, 4.9%, 2.6%, and 20.2% of 1,022 FQ-resistant *Escherichia coli* isolates from humans, animals, and the environment, respectively.

However, these comparisons should be taken with caution since different criteria for the selection of the bacterial isolates were used in these studies. In the present study, the criteria used for selecting these strains included resistance to nalidixic acid, as well as resistance or reduced susceptibility to any, or all of the FQs. These criteria were chosen since *qnr*A1, the first PMQR determinant to be discovered, increased the MIC of nalidixic acid to clinically resistant levels [54] and because a ciprofloxacin MIC of ≥ 0.125 µg/mL is the minimum expected for *Enterobacteriaceae* containing a *qnr* gene [34]. However, it is noteworthy that several *qnr*-positive isolates have increasingly been detected in nalidixic acid-susceptible isolates [51],[55],[56] and so the true prevalence of *qnr* determinants could be underestimated in this study.

The prevalence of the qnr genes were more in *E.coli* and *Salmonella sp*. The lower prevalence of *qnr* genes in *Klebsiella* spp. isolates than in *E. coli* isolates is unlike what was observed in other studies conducted in France [52], USA [34], Spain [53], and China [39]. In France, for example, *qnr* was detected in 0.63% (3/472) and 7% (5/70) among the *E. coli* and *Klebsiella* spp. isolates, respectively. *qnrS* and *qnrB* were detected in six and two isolates, respectively, while *qnrA* was not found in any of the isolates.

The dominance of *qnrB* as the present investigation showed is similar to other studies from Europe [47],[52]. In contrast, *qnrA* gene was dominant in a selected collection of blood culture isolates of *Enterobacteriaceae* resistant to both ciprofloxacin and cefotaxime in UK [57]. Furthermore, *qnrA1* was the most prevalent *qnr* gene in a Spanish study [53] where *qnrA1* was detected in 14 of 305 ESBL-producing enterobacterial isolates whereas only one *qnrS* and no *qnrB* were detected. The implication of results obtained in this study is that, the global phenomenon of PMQR epidemic is becoming a local reality in Nigeria.

## 5. Conclusion

Nigeria is not left out in the global, ravaging emergence of plasmid-mediated fluoroquinolone resistance (PMQR) that precipitates cases of treatment failure. This menace is further compounded by the fact that such resistance has a potential of rapid spread among the bacterial pool. The study revealed a high level of FQ resistance, which was quite significant in samples from human and animal sources. However, a markedly higher Plasmid-mediated FQ resistance was observed among isolates from animal sources than from human sources. Furthermore, these cases of plasmid-mediated resistance were higher among poultry litter isolates than fish pond water isolates. In this study, the quinolone resistance determinants- *qnrA, qnrB* and *qnrS*, were more implicated in conferring the plasmid-mediated FQ resistance and they were found on plasmids.
